# Supplementary material for: Spectroscopic visualization and phase manipulation of chiral charge density waves in 1T-TaS2
Source: Nat Commun. 2023 Apr 19;14:2223. doi: 10.1038/s41467-023-37927-6 (PMC10115830; doi:10.1038/s41467-023-37927-6)
Supplement: Supplementary file 1 — Supplementary Information [file 41467_2023_37927_MOESM1_ESM.pdf]

Supplementary Information for

Spectroscopic Visualization and Phase Manipulation of Chiral Charge Density Waves in 1T-TaS_2

Yan Zhao[1,2,8], Zhengwei Nie[3,4,8], Hao Hong[5], Xia Qiu[2,4,6], Shiyi Han[1], Yue Yu[1], Mengxi Liu[4,6], Xiaohui Qiu[4,6], Kaihui Liu[5], Sheng Meng[3,4,7]*, Lianming Tong[1]*, Jin Zhang[1]

[1]College of Chemistry and Molecular Engineering, Beijing Science and Engineering Center for Nanocarbons, Beijing National Laboratory for Molecular Sciences, Peking University, Beijing 100871, P. R. China

[2] Academy for Advanced Interdisciplinary Studies, Peking University, Beijing 100871, P. R. China

[3] Beijing National Laboratory for Condensed Matter Physics and Institute of Physics, Chinese Academy of Sciences, Beijing 100190, P. R. China

[4] University of Chinese Academy of Sciences, Beijing 100049, P. R. China

[5] State Key Lab for Mesoscopic Physics and Frontiers Science Center for Nano-optoelectronics, Collaborative Innovation Center of Quantum Matter, School of Physics, Peking University, Beijing 100871, P. R. China

[6] CAS Key Laboratory of Standardization and Measurement for Nanotechnology, CAS Center for Excellence in Nanoscience, National Center for Nanoscience and Technology, Beijing 100190, P. R. China

[7] Songshan Lake Materials Laboratory, Dongguan, Guangdong 523808, P. R. China

[8] These authors contributed equally: Yan Zhao, Zhengwei Nie.

*Corresponding author: Sheng Meng (smeng@iphy.ac.cn); Lianming Tong (tonglm@pku.edu.cn)

The supplementary information includes:

I. Symmetry analysis of 1T-TaS_2 and its Raman selection rule

II. Helicity selection rule of 1T-TaS_2

III. Analysis of the SHG response of 1T-TaS_2

IV. First-principle calculations of the energies for different stacking orders

V. Additional data

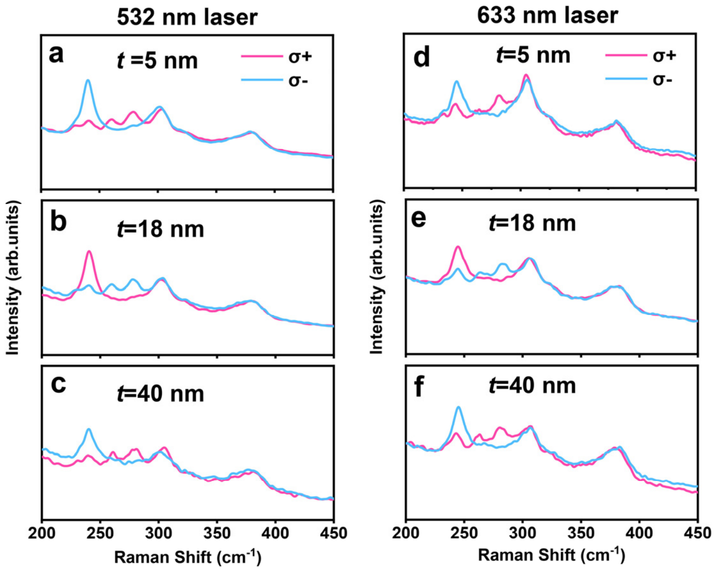

Supplementary Figure 1. Chiral Raman signals for 1T-TaS_2 flakes with different thicknesses. a~c, Chiral Raman spectra of 1T-TaS_2 flakes with the thickness (t) of **a,** t=5 nm, **b,** t=18 nm and **c,** t=40 nm under 532 nm laser excitation. **d~f**, Chiral Raman spectra of 1T-TaS_2 flakes with the thickness of **d,** t=5 nm, **e,** t=18 nm and **f,** t=40 nm under 633 nm laser excitation. The 1T-TaS_2 flake with the thickness of 18 nm show opposite chiral Raman response to the samples with the thickness of 5 nm and 40 nm, corresponding to the two enantiomers of chiral 1T-TaS_2. The samples with opposite chirality in our experiments can be generated during the exfoliating process by turning over the flakes, equivalent to performing a mirror reflection.

Section I. Symmetry analysis of 1T-TaS_2 and the Raman selection rule

The bulk 1T-TaS_2 in the normal state (> 550 K) belongs to the point group of D_{3d}[1]. There are 3 atoms in the unit cell and 9 phonons in total, and the representations of the zone-center normal modes are: $\Gamma=A_{1g}+E_g+2A_{2u}+2E_u$. E_g and A_{1g} modes are Raman active, E_u and A_{2u} modes are infrared-active[2-4]. The E_g mode is doubly degenerate and represents the in-plane atomic vibration, whereas the A_{1g} mode represents the out-of-plane atomic vibration[3]. For the NCCDW and CCDW phase, there are 39 atoms in the unit cell, thus 117 phonons in total. As for their structure symmetry, there still exist controversial opinions. Determined by the interlayer stacking sequence, there are two possible symmetries: trigonal or triclinic[3]. Some researchers think that the structure

remains trigonal and the point group is C_{3i}[4,5], whereas some other researchers report the triclinic symmetry and the point group of C_i[6,7]. For the trigonal case, the normal modes at Γ point are: $19E_g+19A_g+20E_u+20A_u$[2]. For the triclinic case, all the Raman-active modes are with A_g symmetry[3]. To verify the symmetry of the superlattice, we measured the angle-resolved polarized Raman spectra (ARPRS) for the NCCDW and CCDW phase. That is, the polarizers in the incident and collective light paths were fixed to be parallel (VV) or perpendicular (VH) to each other, and the sample orientation was rotated constantly (Supplementary Figure 2a). The ARPRS results for NCCDW and CCDW phases are shown in Supplementary Figure 3 and Supplementary Figure 4, respectively.

Raman selection rule[8] can be calculated by $\mathrm{I}\propto\left|\boldsymbol{\varepsilon}_s^{\dagger}\cdot\mathbf{R}\cdot\boldsymbol{\varepsilon}_i\right|^2$, where $\mathbf{R}$ is Raman tensor, $\boldsymbol{\varepsilon}_i$ and $\boldsymbol{\varepsilon}_s$ are the Jones vectors of incident and Raman scattered light. The forms of Raman tensors for the trigonal and triclinic symmetries are different. For the trigonal system, Raman tensors for E_g and A_{1g} modes are[5,8]:

$$E_g:\ \begin{pmatrix}0 & d & 0\\ d & 0 & e\\ 0 & f & 0\end{pmatrix},\ \begin{pmatrix}d & 0 & -e\\ 0 & -d & 0\\ -f & 0 & 0\end{pmatrix};\ A_{1g}:\ \begin{pmatrix}a & 0 & 0\\ 0 & a & 0\\ 0 & 0 & b\end{pmatrix}.$$

The incident polarized light can be expressed by the Jones vector: $\begin{pmatrix}\sin\theta\\ \cos\theta\\ 0\end{pmatrix}$, where θ is the angle between the polarization direction of the incident light and the orientation of the sample. For the E_g mode, the Raman intensities of the VV and VH configurations are:

$$I_{E_g}^{VV}=\left|(\sin\theta\quad\cos\theta\quad 0)\cdot\begin{pmatrix}0 & d & 0\\ d & 0 & e\\ 0 & f & 0\end{pmatrix}\cdot\begin{pmatrix}\sin\theta\\ \cos\theta\\ 0\end{pmatrix}\right|^2$$

$$+\left|(\sin\theta\quad\cos\theta\quad 0)\cdot\begin{pmatrix}d & 0 & -e\\ 0 & -d & 0\\ -f & 0 & 0\end{pmatrix}\cdot\begin{pmatrix}\sin\theta\\ \cos\theta\\ 0\end{pmatrix}\right|^2=d^2 \qquad (1)$$

$$I_{E_g}^{VH}=\left|(\cos\theta\quad -\sin\theta\quad 0)\cdot\begin{pmatrix}0 & d & 0\\ d & 0 & e\\ 0 & f & 0\end{pmatrix}\cdot\begin{pmatrix}\sin\theta\\ \cos\theta\\ 0\end{pmatrix}\right|^2$$

$$+\left|\begin{pmatrix}\cos\theta & -\sin\theta & 0\end{pmatrix}\cdot\begin{pmatrix}d & 0 & -e\\ 0 & -d & 0\\ -f & 0 & 0\end{pmatrix}\cdot\begin{pmatrix}\sin\theta\\ \cos\theta\\ 0\end{pmatrix}\right|^2 = d^2 \tag{2}$$

For the A_{1g} mode, the Raman intensities under the VV and VH configurations are:

$$I_{A_{1g}}^{VV} = \left|\begin{pmatrix}\sin\theta & \cos\theta & 0\end{pmatrix}\cdot\begin{pmatrix}a & 0 & 0\\ 0 & a & 0\\ 0 & 0 & b\end{pmatrix}\cdot\begin{pmatrix}\sin\theta\\ \cos\theta\\ 0\end{pmatrix}\right|^2 = a^2 \tag{3}$$

$$I_{A_{1g}}^{VH} = \left|\begin{pmatrix}\cos\theta & -\sin\theta & 0\end{pmatrix}\cdot\begin{pmatrix}a & 0 & 0\\ 0 & a & 0\\ 0 & 0 & b\end{pmatrix}\cdot\begin{pmatrix}\sin\theta\\ \cos\theta\\ 0\end{pmatrix}\right|^2 = 0 \tag{4}$$

Thus the Raman intensities of E_g and A_{1g} modes for both VV and VH configurations do not vary with the sample orientation for the trigonal symmetry.

For the triclinic system, the Raman tensor for A_{1g} mode is[8]:

$$\begin{pmatrix}a & d & e\\ d & b & f\\ e & f & c\end{pmatrix}.$$

And the Raman intensities under the VV and VH configurations are:

$$I_{A_g}^{VV} = \left|\begin{pmatrix}\sin\theta & \cos\theta & 0\end{pmatrix}\cdot\begin{pmatrix}a & d & e\\ d & b & f\\ e & f & c\end{pmatrix}\cdot\begin{pmatrix}\sin\theta\\ \cos\theta\\ 0\end{pmatrix}\right|^2$$

$$= (a\sin^2\theta + 2d\sin\theta\cos\theta + b\cos^2\theta)^2 \tag{5}$$

$$I_{A_g}^{VH} = \left|\begin{pmatrix}\cos\theta & -\sin\theta & 0\end{pmatrix}\cdot\begin{pmatrix}a & d & e\\ d & b & f\\ e & f & c\end{pmatrix}\cdot\begin{pmatrix}\sin\theta\\ \cos\theta\\ 0\end{pmatrix}\right|^2$$

$$= [d(\cos^2\theta - \sin^2\theta) + (a-b)\sin\theta\cos\theta]^2 \tag{6}$$

It is obvious that the Raman intensities for the VV and VH configurations are both relevant to θ.

According to the theoretical analysis above, the calculated Raman selection rule for the trigonal symmetry is consistent with the experimental results shown in Supplementary Figure 3 and Supplementary Figure 4, thus we conclude that the structure symmetry for the NCCDW and CCDW phase of layered 1T-TaS_2 should be trigonal.

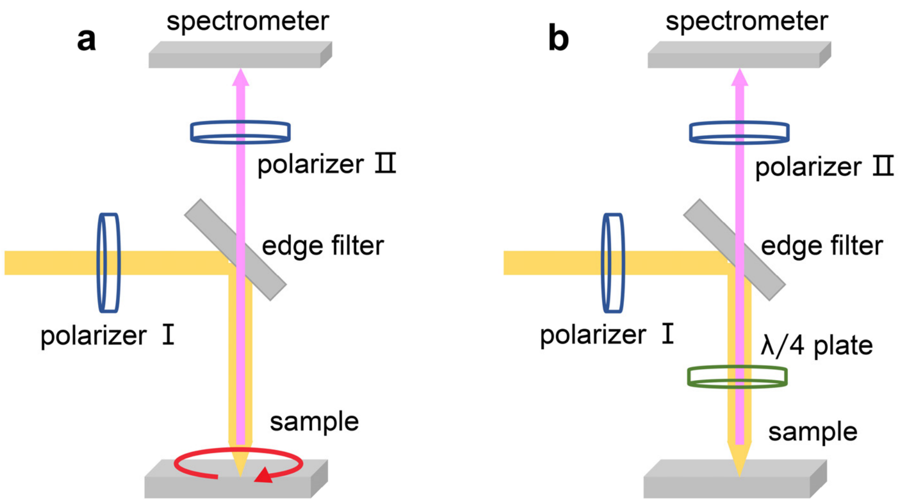

Supplementary Figure 2. Schematics of the optical setups for the linearly and circularly polarized Raman spectra. **a**, Angle-resolved linearly polarized Raman spectroscopy, the sample orientation is rotated constantly. **b**, Helicity-resolved Raman spectroscopy, the circularly polarized light is generated by the quarter wave (λ/4) plate.

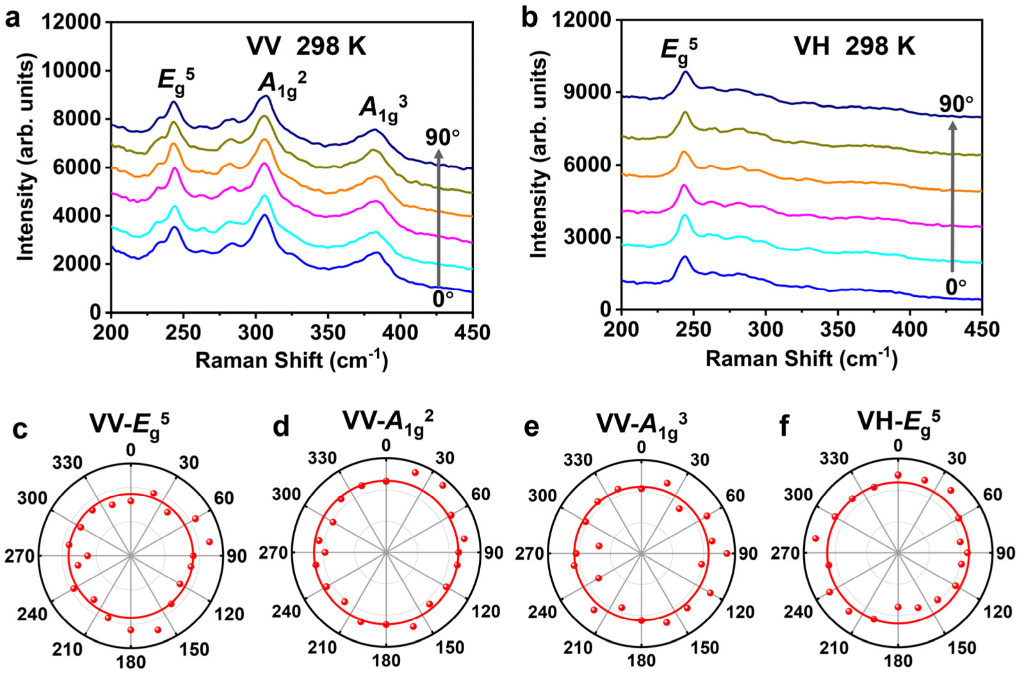

Supplementary Figure 3. Angle-resolved polarized Raman spectra of layered 1T-TaS_2 flakes at 298 K (NCCDW phase). **a** and **b**, ARPRS for **a**, co-polarized (VV) and **b**, cross-polarized (VH) configurations with the polarization angle θ ranging from 0°

to 90°. **c~f**, Polar plots of the Raman intensities with the variation of θ for the **c**, E_g^5 mode; **d**, A_{1g}^2 mode; **e**, A_{1g}^3 modes under the VV configuration and **f**, E_g^5 mode under the VH configuration.

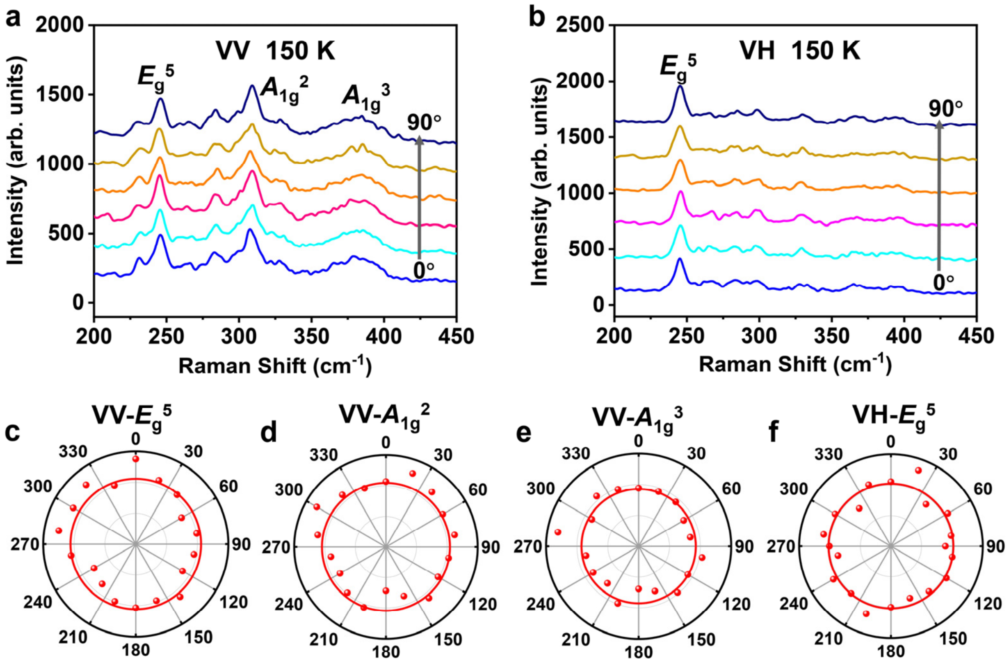

Supplementary Figure 4. Angle-resolved polarized Raman spectra of layered 1T-TaS_2 flakes at 150 K (CCDW phase). a and **b**, ARPRS for VV and VH configurations with the polarization angle θ ranging from 0° to 90°. **c~f**, Polar plots of the Raman intensities with the variation of θ for the **c**, E_g^5 mode; **d**, A_{1g}^2 mode; **e**, A_{1g}^3 mode under the VV configuration and **f**, E_g^5 mode under the VH configuration.

Section II. Helicity selection rule of 1T-TaS_2

The helicity selection rule can be deduced by $I \propto \left|\boldsymbol{\sigma}_s^\dagger \cdot \mathbf{R} \cdot \boldsymbol{\sigma}_i\right|^2$, where $\boldsymbol{\sigma}_i$ and $\boldsymbol{\sigma}_s$ are the Jones vectors of incident and Raman scattered light, and can be expressed by $\frac{1}{\sqrt{2}}\begin{pmatrix}1\\ i\\ 0\end{pmatrix}$ and $\frac{1}{\sqrt{2}}\begin{pmatrix}1\\ -i\\ 0\end{pmatrix}$ for left-handed and right-handed circularly polarized light, respectively. The Raman intensities of E_g and A_{1g} modes under σ+σ+ and σ+σ- configurations are:

$$I_{E_g}^{\sigma+\sigma+} \propto \left| \frac{1}{\sqrt{2}} (1 \quad -i \quad 0) \cdot \begin{pmatrix} 0 & d & 0 \\ d & 0 & e \\ 0 & f & 0 \end{pmatrix} \cdot \frac{1}{\sqrt{2}} \begin{pmatrix} 1 \\ i \\ 0 \end{pmatrix} \right|^2$$

$$+ \left| \frac{1}{\sqrt{2}} (1 \quad -i \quad 0) \cdot \begin{pmatrix} d & 0 & -e \\ 0 & -d & 0 \\ -f & 0 & 0 \end{pmatrix} \cdot \frac{1}{\sqrt{2}} \begin{pmatrix} 1 \\ i \\ 0 \end{pmatrix} \right|^2 = 0 \quad (7)$$

$$I_{E_g}^{\sigma+\sigma-} \propto \left| \frac{1}{\sqrt{2}} (1 \quad i \quad 0) \cdot \begin{pmatrix} 0 & d & 0 \\ d & 0 & e \\ 0 & f & 0 \end{pmatrix} \cdot \frac{1}{\sqrt{2}} \begin{pmatrix} 1 \\ i \\ 0 \end{pmatrix} \right|^2$$

$$+ \left| \frac{1}{\sqrt{2}} (1 \quad i \quad 0) \cdot \begin{pmatrix} d & 0 & -e \\ 0 & -d & 0 \\ -f & 0 & 0 \end{pmatrix} \cdot \frac{1}{\sqrt{2}} \begin{pmatrix} 1 \\ i \\ 0 \end{pmatrix} \right|^2 = 2d^2 \quad (8)$$

$$I_{A_{1g}}^{\sigma+\sigma+} \propto \left| \frac{1}{\sqrt{2}} (1 \quad -i \quad 0) \cdot \begin{pmatrix} a & 0 & 0 \\ 0 & a & 0 \\ 0 & 0 & b \end{pmatrix} \cdot \frac{1}{\sqrt{2}} \begin{pmatrix} 1 \\ i \\ 0 \end{pmatrix} \right|^2 = a^2 \quad (9)$$

$$I_{A_{1g}}^{\sigma+\sigma-} \propto \left| \frac{1}{\sqrt{2}} (1 \quad i \quad 0) \cdot \begin{pmatrix} a & 0 & 0 \\ 0 & a & 0 \\ 0 & 0 & b \end{pmatrix} \cdot \frac{1}{\sqrt{2}} \begin{pmatrix} 1 \\ i \\ 0 \end{pmatrix} \right|^2 = 0 \quad (10)$$

Thus the E_g mode is helicity-changed and the A_{1g} mode is helicity-conserved.

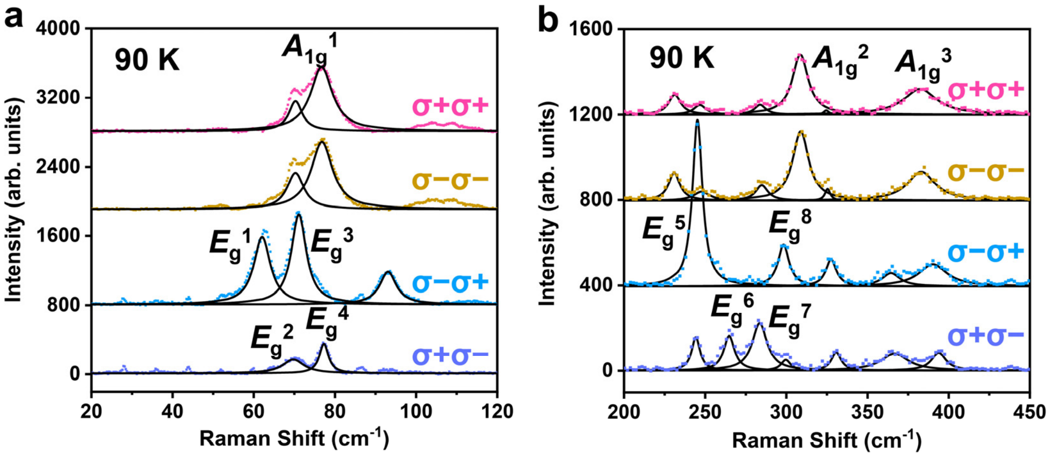

Supplementary Figure 5. HRRS for the CCDW phase at 90 K. **a**, HRRS for the co-polarized (σ+σ+, σ-σ-) and cross-polarized (σ+σ-, σ-σ+) configurations at lower frequency (20~120 cm^{-1}). We have marked the peaks that also appear for the NCCDW phase, and the peak positions show blueshift compared with that of the NCCDW phase. Two A_{1g} modes and five E_g modes can be identified here. **b**, HRRS at higher frequency (200~450 cm^{-1}). Almost all the E_g modes show apparent chiral response, while the A_{1g}

modes remain the same for the σ+ and σ- excitation, which is consistent with the NCCDW phase.

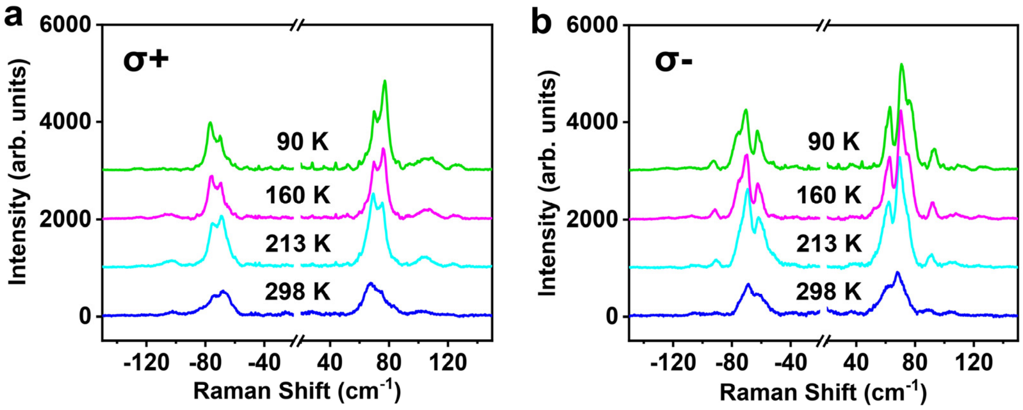

Supplementary Figure 6. The low-frequency Raman modes of layered 1T-TaS_2 with the variation of temperature across the NCCDW and CCDW phases. a and **b,** The variation of low-frequency Raman spectra under **a**, σ+ and **b**, σ- excitation. There shows apparent difference in the low-frequency Raman modes under σ+ and σ- excitation at various temperatures.

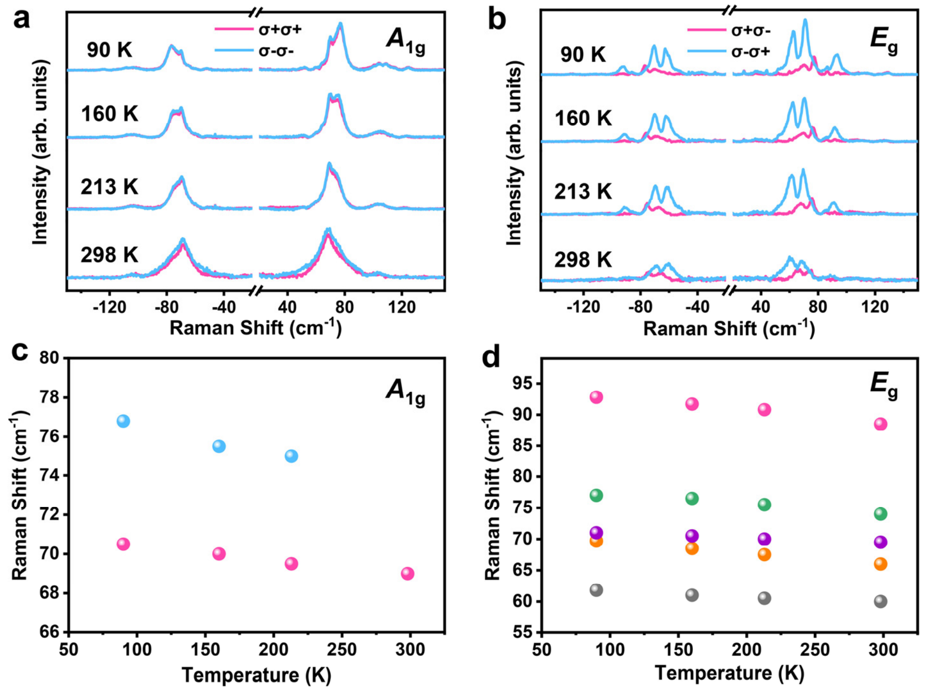

Supplementary Figure 7. The assignment of the low-frequency Raman modes and

their peak positions with the variation of temperature. a, Raman spectra under the σ+σ+ and σ-σ- configurations at various temperatures, where the A_{1g} modes are Raman active. There is a broad A_{1g} Raman peak at 298 K, which has been identified to be the amplitude mode corresponding to the collective excitations of the CDW[9,10]. With the lowering of temperature, the broad peak evolves into two Raman modes with A_{1g} symmetry. **b**, Raman spectra for the σ+σ- and σ-σ+ configurations under which the E_g modes are Raman active. **c**, The peak positions of the Stokes A_{1g} Raman modes in **a** as a function of temperature. **d**, The variation of peak positions of the five Stokes E_g Raman modes in **b** as a function of temperature.

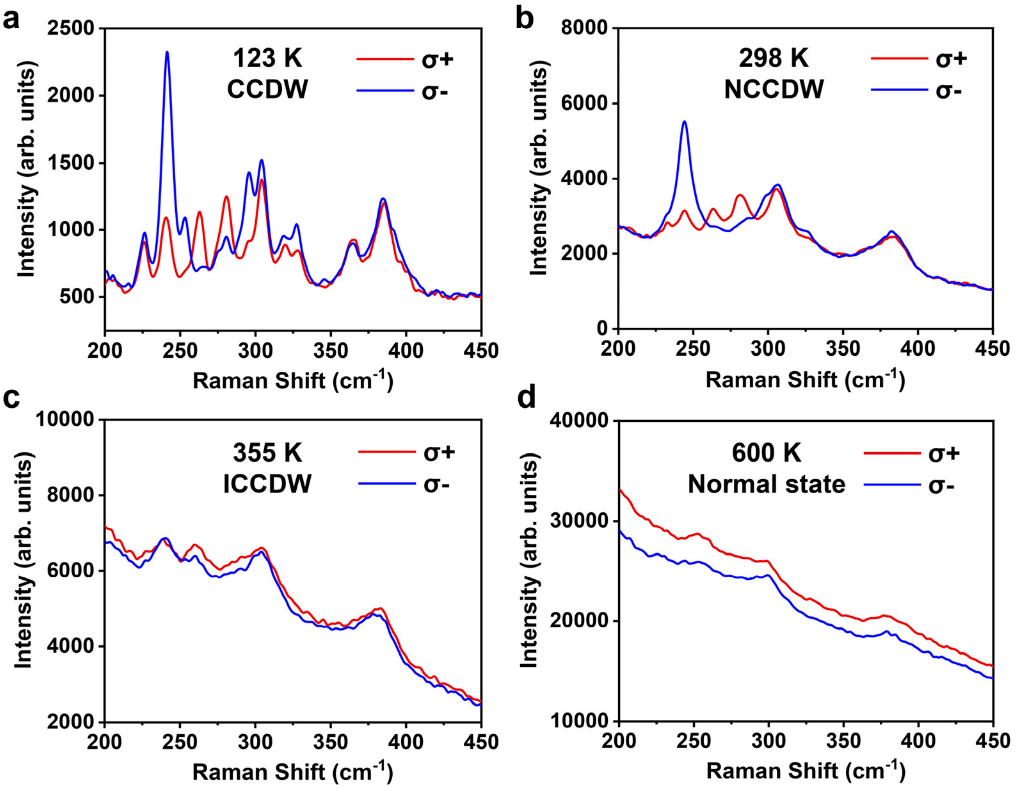

Supplementary Figure 8. Chiral Raman spectra of different phases with the variation of temperature. a, Chiral Raman spectra for the commensurate (C) CDW phase at 123 K. **b**, Chiral Raman spectra for the nearly commensurate (NC) CDW state at 298 K. **c**, Chiral Raman spectra for the incommensurate (IC) CDW state at 355 K. The Raman intensities decrease because of the screening effect of the free carriers[2]. **d**, Chiral Raman spectra for the undistorted phase at 600 K. Both the Raman intensities

and the chiral Raman response are weakened for the ICCDW phase and the undistorted phase. The strong background was previously reported to be a continuum of electron-hole excitations[5].

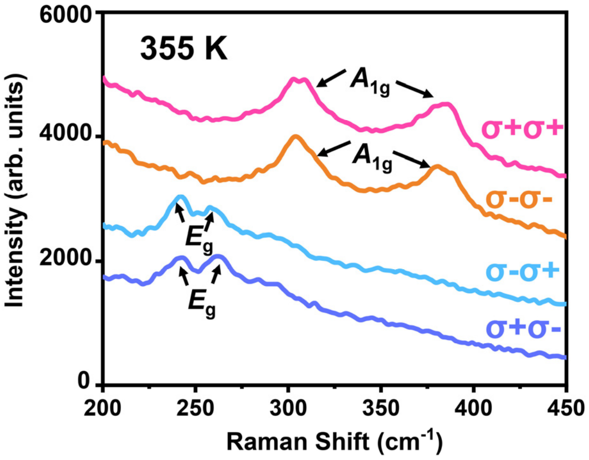

Supplementary Figure 9. HRRS for the ICCDW phase (T=355 K) of 1T-TaS$_2$. The A_{1g} modes remain the same for σ+σ+ and σ-σ- configurations. More importantly, the E_g modes in σ-σ+ and σ+σ- configurations are also the same, reflecting that the chiral Raman response vanishes for the ICCDW phase.

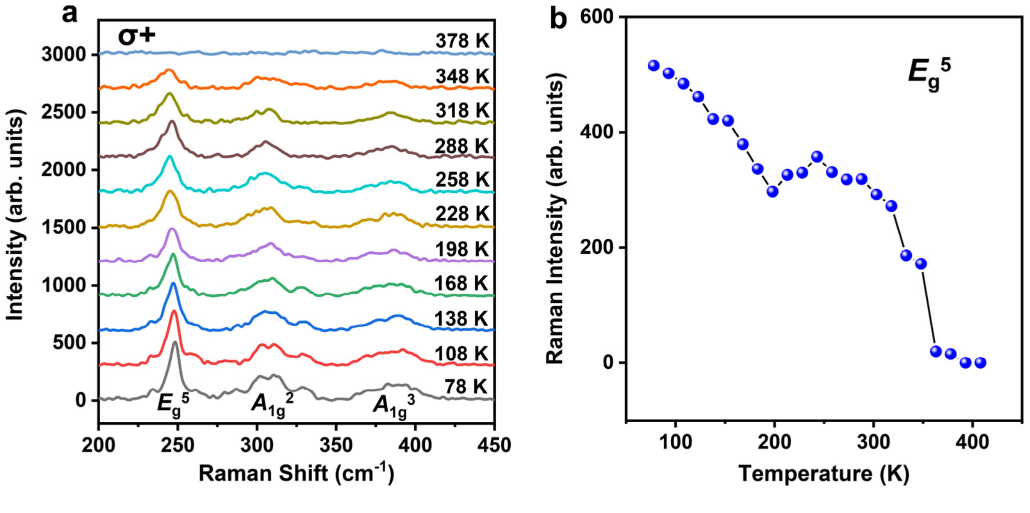

Supplementary Figure 10. Temperature-dependent Raman spectra of 1T-TaS$_2$. a, Raman spectra under σ+ excitation with the variation of temperature. **b**, Temperature-dependent Raman intensities of E_g^5 mode. The sharp changes of Raman intensities at approximately 200 K and 350 K indicate the NCCDW-CCDW and ICCDW-NCCDW

phase transitions, respectively.

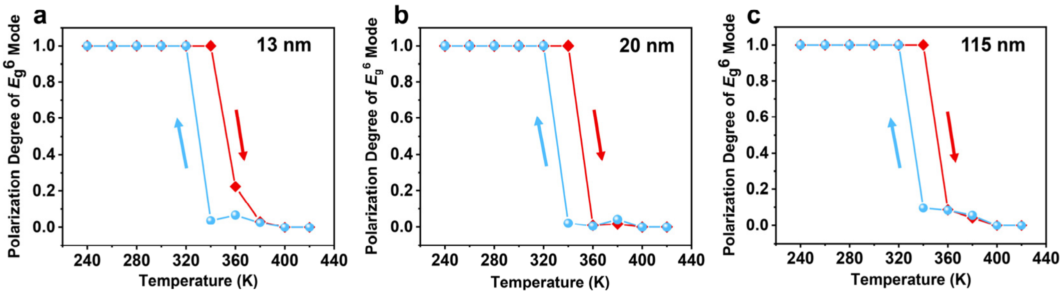

Supplementary Figure 11. Hysteresis behavior of chiral Raman response. Variation of circular polarization degree ($\rho = (I_{\sigma-} - I_{\sigma+})/(I_{\sigma-} + I_{\sigma+})$) of E_g^6 mode for 1T-TaS_2 flakes with the thickness of **a**, 13 nm; **b**, 20 nm; **c**, 115 nm in the heating (red) and cooling (blue) processes. For NCCDW phase, the E_g^6 mode shows clear chiral Raman response, which only appears in σ- polarized excitation with $\rho = 1$. Whereas for the ICCDW phase, the chiral Raman response disappears with $\rho = 0$. Thus the hysteresis behavior of chiral Raman response is consistent with the NCCDW-ICCDW transition process, and the thickness dependence for the hysteresis behavior in the NCCDW-ICCDW transition is somewhat robust, which is consistent with the previous report[11].

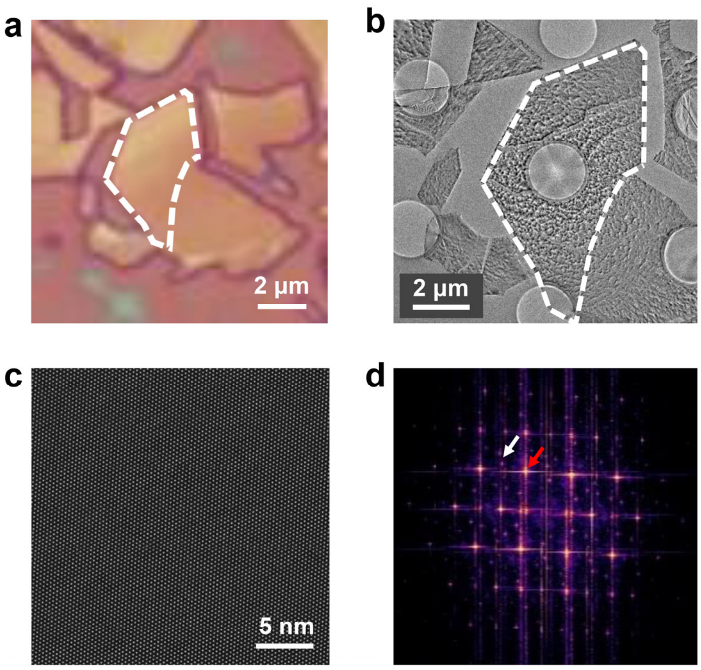

Supplementary Figure 12. The preparation of 1T-TaS_2 samples for transmission electron microscopy (TEM) characterization. a, Optical picture of the mechanically

exfoliated 1T-TaS_2 flake with a thickness of approximately 15 nm on 300 nm SiO_2/Si substrate. **b**, The TEM image of the transferred 1T-TaS_2 flake on SiN_x grid. The sample region illustrated by white dotted lines corresponds to that in **a**. **c**, The atomic-resolution scanning transmission electron microscopy-annular dark field (STEM-ADF) image of the 1T-TaS_2 flake. **d**, The corresponding fast Fourier transform (FFT) pattern of **c**. The red and white arrows indicate the primary spots and the superlattice spots, respectively. The measurements are performed at room temperature.

Section III. Analysis of the SHG response of 1T-TaS_2

Nonlinear optical effect serves as a powerful method to identify the crystalline symmetry and explore the symmetry breakings[12-14]. It has been widely reported that chiral materials may exhibit SHG response[15-17]. The theory for the second-order nonlinear optics of chiral materials is based on electric-dipole approximation of the light-matter interaction and the analysis of the nonlinear optical polarizability. The intensity of SHG is related to the effective second-order polarizability $\mathbf{P}_{(2\omega)}$, which can be expressed as[18]:

$$\boldsymbol{P}_{(2\omega)} = \chi_{(2\omega)}\boldsymbol{E}\boldsymbol{E} + \alpha\{\boldsymbol{E}\cdot\boldsymbol{\nabla}\}\boldsymbol{E} + \beta\boldsymbol{E}\{\boldsymbol{\nabla}\cdot\boldsymbol{E}\} + \left(\frac{i2v}{c}\right)\gamma\{\boldsymbol{E}\times\boldsymbol{B}\} \qquad (11)$$

E and **B** represent the effective electric and magnetic field of the light. $\chi_{(2\omega)}$ is the second-order susceptibility. The first term in the equation represents the electric dipole effect, while the second and third terms represent the quadrupolar effect, and the last term is generated by the magnetic dipole effect. According to equation above, the SHG response can be contributed by the electric dipole, quadrupole, and magnetic dipole effects. The last three terms could be much smaller than the electric dipole term, thus the SHG intensity can be regarded to be proportional to the second-order susceptibility in most cases. And the second-order susceptibility is nonzero only if the material has no inversion symmetry.

1T-TaS_2 in the undistorted phase retains inversion symmetry with the point group of D_{3d}, and the electric dipole contribution to SHG response is forbidden. For the NCCDW and CCDW phases, however, the mirror symmetry is broken, resulting in the

remarkable SHG signal, as has been reported previously[19,20].

For chiral materials, there may appear intensity difference for the SHG signal under excitation of left-handed or right-handed circularly polarized light, which is called the second-harmonic generation circular-dichroism (SHG-CD) effect[16,18,21]. The intensity can be deduced as follows:

The incident polarized light can be expressed by the superposition of *s*- and *p*-polarized light,

$$\boldsymbol{e}E_0 = \boldsymbol{e}_s E_s + \boldsymbol{e}_p E_p. \tag{12}$$

Where **e**, $\mathbf{e}_s$ and $\mathbf{e}_p$ are the polarization vectors. When the incident light passes through a quarter wave plate, the amplitudes of *s*- and *p*-polarized light can be expressed by[22]

$$E_s = E_0 \sin\varphi \cos\varphi\,(1 - i), \tag{13}$$

$$E_p = E_0(\sin^2\varphi + i\cos^2\varphi). \tag{14}$$

Where φ is the rotation angle of the quarter wave plate. $\varphi = \pi/2$ and $\varphi = -\pi/2$ represent the right- and left-handed circularly polarized light, respectively.

The SHG intensity can be expressed by[23,24]

$$I_{2\omega} = \left|fE_p{}^2 + gE_s{}^2 + hE_sE_p\right|^2 \tag{15}$$

where *f*, *g* and *h* are the items related to the second-order polarizability.

SHG-CD effect can be evaluated by anisotropy factor g_{SHG-CD} as shown below[24]:

$$g_{SHG-CD} = \frac{2(I_{\sigma+} - I_{\sigma-})}{I_{\sigma+} + I_{\sigma-}} = \frac{4Im(g * h) + 4Im(h * f)}{|f|^2 + |g|^2 + |h|^2 - 2Re(f * g)} \tag{16}$$

It can be deduced that the SHG intensity will show difference for the right- and left-handed circularly polarized excitation when there exist a phase difference between the coefficients *f*, *g* and *h*[24].

The previous theory pointed out that the electric dipole should not contribute to SHG-CD effect, and the contributions of magnetic dipole and quadrupole effects would also be small, resulting in the weak SHG-CD signal for chiral materials[25]. Till now, the remarkable SHG-CD effect has been reported in chiral molecule solution and films[18], chiral lead halide perovskite[15,16] and so on. The enhanced SHG-CD effect in chiral molecules at the air/water interface has been attributed to the orientation and aggregation of the molecules, under which the CD can be electric dipole-allowed[17,18].

However, more study and understanding of the origin of the enhanced SHG-CD signal is still needed. In this work, we suppose that the prominent SHG-CD effect observed in 1T-TaS_2 may be related to the periodic charge density and the modulated electronic states, which deserves further study and may arouse the research interest in the novel CDW-related nonlinear optical properties.

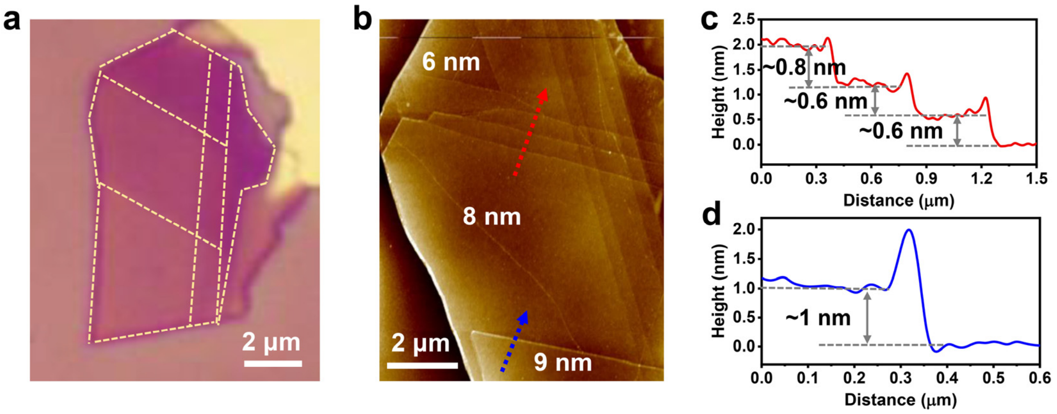

Supplementary Figure 13. The mechanically exfoliated 1T-TaS_2 flakes for exploring the chirality switching phenomenon. a, Optical picture of the multilayer 1T-TaS_2 flake. The dotted lines illustrate the zones with different thicknesses. **b**, AFM image of the multilayer 1T-TaS_2 flake, which shows a clear step-terrace morphology. **c** and **d**, The height profiles along the **c**, red and **d**, blue arrows in **b**. The measured interlayer spacing values (c) are close to the distance $c \approx 0.59$ nm reported in literature[26,27]. The relatively larger spacing of 0.8 nm and 1 nm may come from the wrinkle or strain between layers. The sharp increase of the height at the edge may come from the curl or wrinkle.

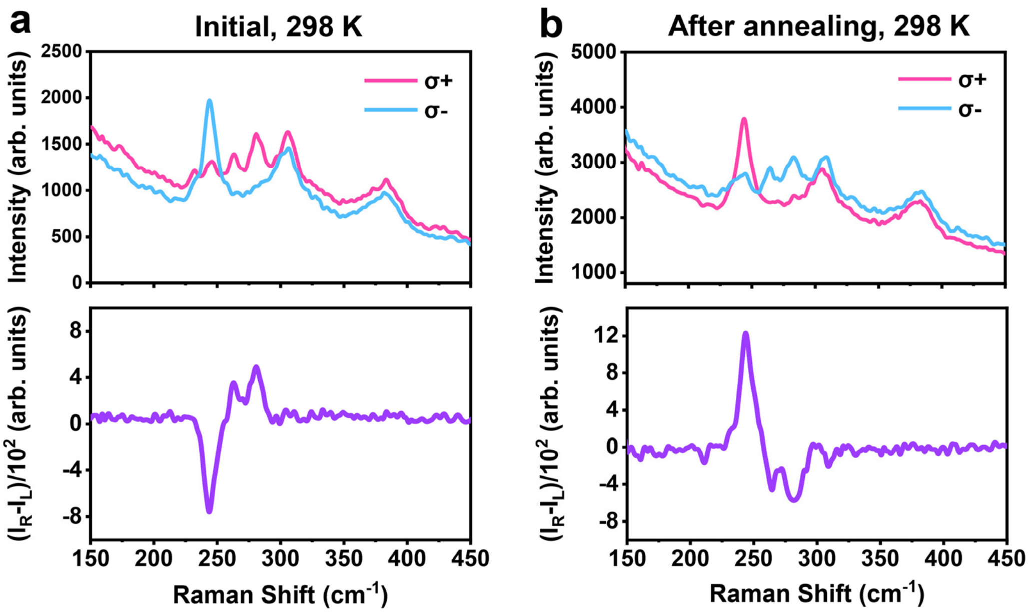

Supplementary Figure 14. Chiral Raman spectra during the chirality switching process. a, Initial chiral Raman spectra at 298 K. **b**, Chiral Raman spectra after the annealing process, and the chirality is reversed.

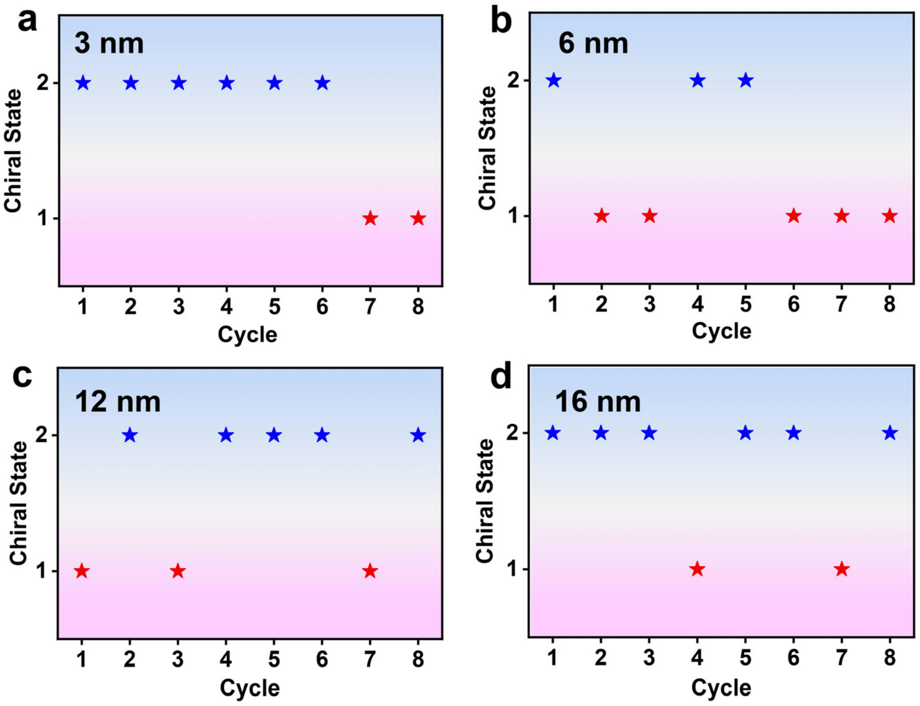

Supplementary Figure 15. The reversible chirality switching during multiple temperature variation cycles. The chiral states of 1T-TaS_2 flakes with the thickness of

a, 3 nm; **b**, 6 nm; **c**, 12 nm; **d**, 16 nm during annealing cycles. For simplicity, the chiral state here is defined according to the relative intensity of E_g^5 mode ($\Delta = I^{\sigma+}_{E_g{}^5} - I^{\sigma-}_{E_g{}^5}$) in the chiral Raman spectra. Chiral state 1: $\Delta > 0$ (indicated by the pink star symbol and shading); Chiral state 2: $\Delta < 0$ (indicated by the blue star symbol and shading). It is apparent that the chirality switching between two equivalent chiral phases is random for 1T-TaS_2 samples with different thicknesses.

Section IV. First-principle calculations of the energies for different stacking orders

The stacking orders can affect the electronic structures of 1T-TaS_2, and may determine it to be either metallic or insulating[28,29]. We first discuss the stacking order of 1T-TaS_2 layers with the same chirality. The alignment of 1T-TaS_2 layers in CCDW phase can be described by the stacking vector $\mathbf{T}_S$, which is defined by the relative position of the central Ta sites (labeled as A) in successive layers. If we do not consider the dimerization of 1T-TaS_2 layers, then there are five stacking orders between adjacent layers[26]: $\mathbf{T}_S$=**c** (A→A), $\mathbf{T}_S$=**a**+**c** (A→B), $\mathbf{T}_S$=-**a**+**c** (A→C), $\mathbf{T}_S$=2**a**+**c** (A→D), $\mathbf{T}_S$=-2**a**+**c** (A→E), which can be named as the A, B, C, D and E stacking correspondingly (Supplementary Figure 16). When the paired stacking of 1T-TaS_2 layers is considered, then every two layers can shift with a selective $\mathbf{T}_S$, generating other four kinds of stacking orders named A-B, A-C, A-D, A-E. We listed the calculated interlayer binding energies (E_{ib}) and total energies (E_{total}) of these possible stacking orders in Supplementary Table 1, with $E_{ib} = (\sum E_{layer} - E_{total})/N_{cell}$. Usually, the typical interlayer binding energy of graphite is about ~100 meV/atom[30]. Our DFT results of binding energy (4.4 eV/star≈113 meV/atom) is very close to this number, indicating it is a reasonable value. It can be seen that the paired A-E stacking is the most stable one with the highest binding energy for the stacking patterns with the same chirality, leading to the lowest total energy of the system (the total energies of other stacking orders are given with respect to the most stable A-E stacking of which E_{total} is set to be 0), which is consistent with the previously reported results[26].

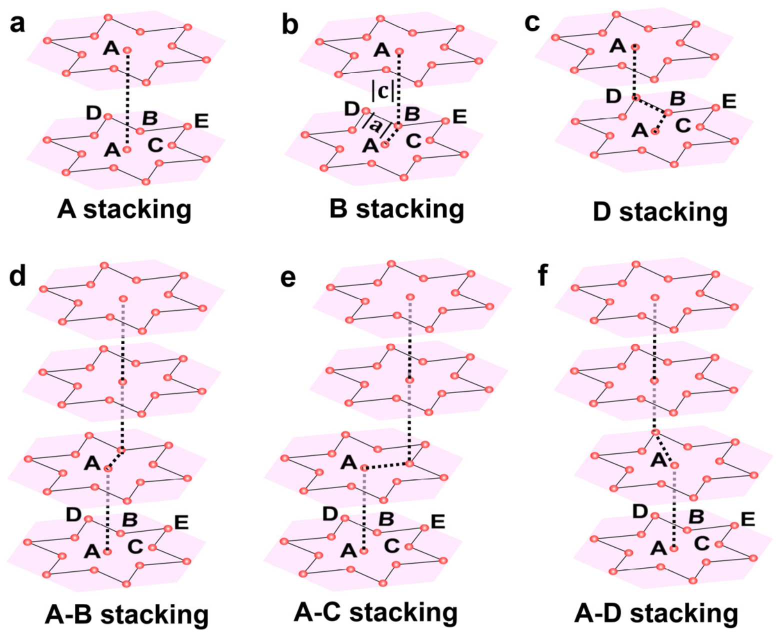

Supplementary Figure 16. Schematics of the stacking patterns for 1T-TaS_2 layers with the same chirality. a-c, The stacking patterns of **a**, A stacking; **b**, B stacking; **c**, D stacking without considering dimerization between layers. **|a|** and **|c|** in **b** mean the lattice constant and interlayer distance, respectively. **d-f**, The paired stacking configurations of **d**, A-B stacking; **e**, A-C stacking; **f**, A-D stacking.

Supplementary Table 1. Calculated energies of several stacking configurations for 1T-TaS_2 layers with the same chirality. The total energy is given with respect to the most stable A-E stacking.

Stacking Order	Total Energy (meV/star)	Binding Energy (eV/star)	Stacking Order	Total Energy (meV/star)	Binding Energy (eV/star)
**A**	47.7	4.411	**A-B**	44.4	4.415
**B**	47.0	4.417	**A-C**	38.7	4.417
**C**	34.4	4.428	**A-D**	34.5	4.421
**D**	58.6	4.410	**A-E**	0	4.454
**E**	3.4	4.453			

For the opposite-chirality stacking, similarly, we can also list the stacking patterns according to the relative shift between successive layers with opposite chirality. Taking opposite-chirality dimerization into account, there are two marks to characterize 3D configurations, defined as $\mathbf{T}_{S1}$ and $\mathbf{T}_{S2}$. The former designates the relative position connecting two opposite-chirality stars in a dimer, while the latter designates the relative position connecting two stars between dimers (Supplementary Figure 17). For example, A-E$'$ stacking means that the central Ta site of the star-of-David in one of the two adjacent layers are aligned with $\mathbf{T}_{S1}$=**c**, and the Ta in the other layer is stacked with a relative displacement $\mathbf{T}_{S2}$=-2**a**+**c**. The notation X$'$ is used to differentiate from X (X=A, B, C, D, E) used for the same-chirality stacking. We list the calculated binding energies in Supplementary Table 2, among which the A-E$'$ stacking shows the highest binding energy and is the most stable one among all the listed stacking configurations. It's worth noting that the energy difference of about 0.5 eV/star accounts for ~11% of the interlayer binding energy of α/α stacking.

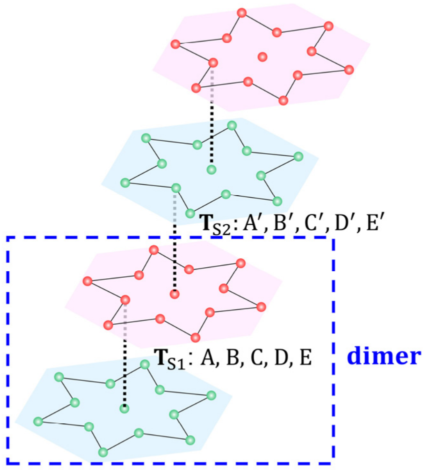

Supplementary Figure 17. Schematic of the stacking pattern for 1T-TaS_2 layers with opposite chirality.

Supplementary Table 2. The total energy and binding energy of several stacking configurations for 1T-TaS_2 layers stacked with opposite chirality. The values of total energy are given with respect to that of A-E stacking shown in Supplementary Table 1.

Stacking Order	Total Energy (meV/star)	Binding Energy (eV/star)	Stacking Order	Total Energy (meV/star)	Binding Energy (eV/star)	Stacking Order	Total Energy (meV/star)	Binding Energy (eV/star)
**A-A′**	592.0	3.873	**B-E′**	586.9	3.878	**D-D′**	612.3	3.853
**A-B′**	571.2	3.894	**C-A′**	595.5	3.869	**D-E′**	595.9	3.871
**A-C′**	590.4	3.877	**C-B′**	566.0	3.904	**E-A′**	568.6	3.894
**A-D′**	573.8	3.891	**C-C′**	585.2	3.887	**E-B′**	574.9	3.890
**A-E′**	554.5	3.908	**C-D′**	590.5	3.877	**E-C′**	582.0	3.882
**B-A′**	564.7	3.9	**C-E′**	556.6	3.910	**E-D′**	598.6	3.868
**B-B′**	595.6	3.870	**D-A′**	570.2	3.894	**E-E′**	592.5	3.870
**B-C′**	571.3	3.899	**D-B′**	595.4	3.869			
**B-D′**	591.0	3.875	**D-C′**	584.6	3.882			

The definition of *g(r)*:

In statistical mechanics, the radial distribution function $g(r)$ describes how density varies as a function of distance from a reference particle. Consider a system of *N* atoms with density ρ, then $\rho g(r)dr$ is the probability of observing a second atom in dr given that there is an atom at the origin of r. It gives

$$\int_0^\infty \rho g(r) 4\pi r^2 dr = N - 1 \tag{17}$$

The function $g(r)$ can also be thought of as the factor that multiplies the bulk density ρ to give a local density $\rho(r) = \rho g(r)$ about some fixed particles. It is also called a correlation function, since if the particles were independent of each other, $\rho(r)$ would simply equal ρ, so the factor $g(r) \approx 1$. Usually in a solid the atoms are arranged in a regular repeating order, and this lead to sharp peaks in the $g(r)$ curves.

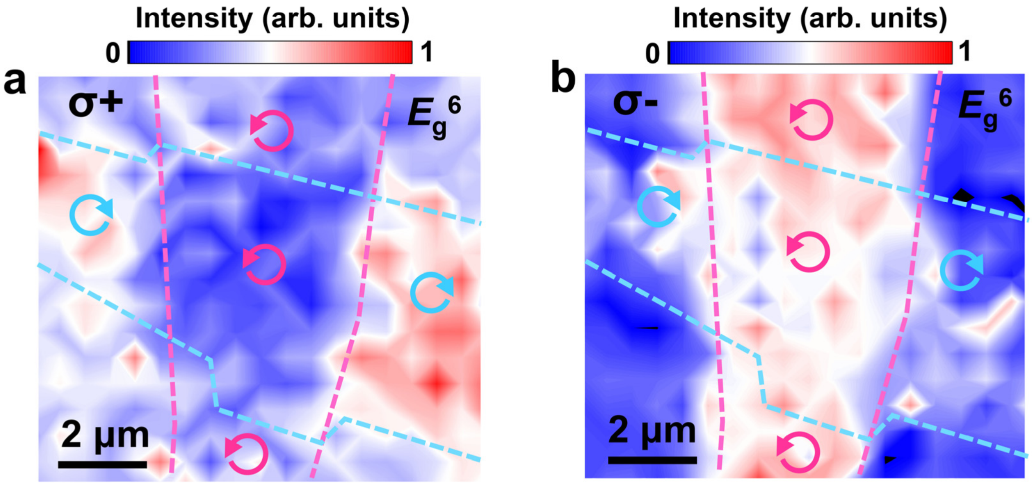

Supplementary Figure 18. The generation of in-plane chiral homostructure. Raman intensity mapping image of the E_g^6 mode under **a**, σ+ and **b**, σ- excitation after the annealing process. The blue and pink arrows indicate the opposite chirality of several parts in the sample.

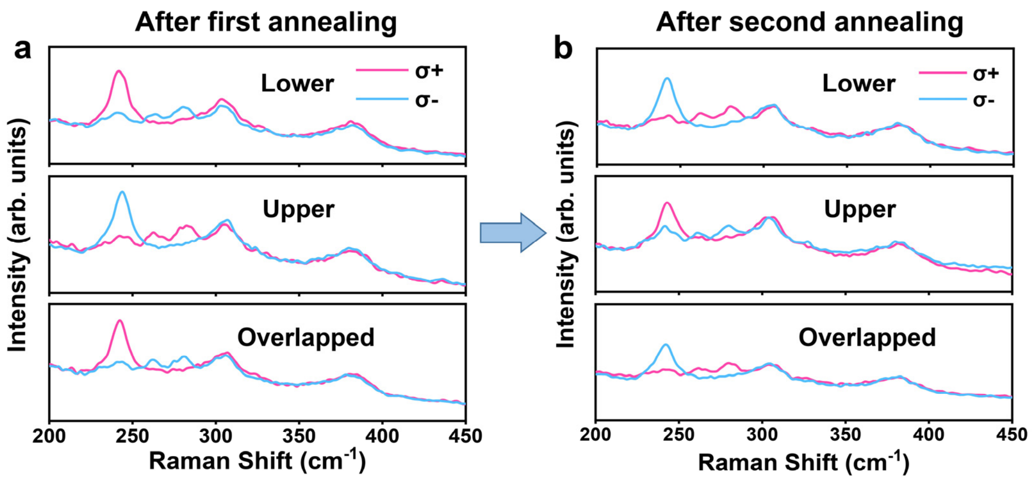

Supplementary Figure 19. The variation of the chirality of lower flake, upper flake and overlapped zone in the homostructure. a, Chiral Raman spectra of the homostructure after the first annealing process (selected from Fig. 5d). **b**, Chiral Raman spectra of the homostructure after further annealing above 350 K. Both the lower and upper flake reverse their chirality. The upper flake on the substrate and the part in the overlapped zone show opposite chiral Raman response, indicating that they switch the chirality independently.

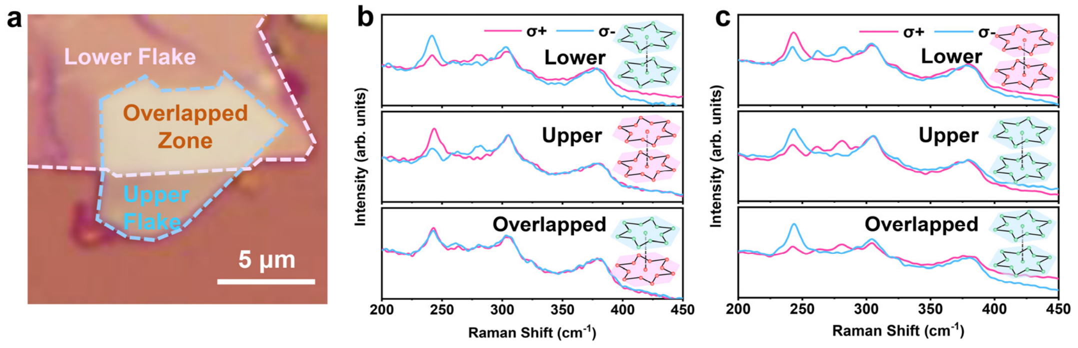

Supplementary Figure 20. The in-plane chiral homostructure generated in the lower flake. a, Optical picture of another α/β 1T-TaS_2 homostructure. **b**, Chiral Raman spectra of the initial sample. The lower and upper flakes show opposite chirality, and the chiral Raman response of the overlapped zone vanishes, as illustrated by the stacking schematic diagram inset (green and pink stars represent opposite chirality). **c**, Chiral Raman spectra of the sample after annealing (473 K, 2 hours). The chirality of both the lower and upper flakes are reversed after the annealing process, and there appears chiral Raman response for the overlapped zone, which is the same as the upper flake. Thus the in-plane chiral homostructure is generated in the lower flake.

Supplementary References

1. Sai-Halasz, G. A. & Perry, P. B. Charge density waves effects on the phonon modes of 1T-TaS_2. *Solid State Commun.* **21**, 995-997 (1977).
2. Gasparov, L. V. *et al.* Phonon anomaly at the charge ordering transition in 1T-TaS_2. *Phys. Rev. B* **66**, 094301 (2002).
3. Albertini, O. R. *et al.* Zone-center phonons of bulk, few-layer, and monolayer 1T-TaS_2: Detection of commensurate charge density wave phase through Raman scattering. *Phys. Rev. B* **93**, 214109 (2016).
4. Uchida, S. & Sugai, S. Infrared and Raman studies on commensurate CDW states in transition metal dichalcogenides. *Physica B+C* **105**, 393-399 (1981).
5. Mijin, S. D. *et al.* Probing charge density wave phases and the Mott transition in 1T-TaS_2 by inelastic light scattering. *Phys. Rev. B* **103**, 245133 (2021).
6. Duffey, J. R., Kirby, R. D. & Coleman, R. V. Raman scattering from 1T-TaS_2. *Solid*

State Commun. **20**, 617-621 (1976).

7. Kennedy, R. J. & Clayman, B. P. Far-infrared studies of the commensurate charge-density wave of 1T-TaS_2. *Phys. Rev. B* **29**, 851-856 (1984).
8. Loudon, R. The Raman effect in crystals. *Adv. Phys.* **50**, 813-864 (2001).
9. Wen, W., Zhu, Y., Dang, C., Chen, W. & Xie, L. Raman spectroscopic and dynamic electrical investigation of multi-state charge-wave-density phase transitions in 1T-TaS_2. *Nano Lett.* **19**, 1805-1813 (2019).
10. McMillan, W. L. Landau theory of charge-density waves in transition-metal dichalcogenides. *Phys. Rev. B* **12**, 1187-1196 (1975).
11. Yoshida, M. *et al.* Controlling charge-density-wave states in nano-thick crystals of 1T-TaS_2. *Sci. Rep.* **4**, 7302 (2014).
12. Harter, J. W., Zhao, Z. Y., Yan, J.-Q., Mandrus, D. G. & Hsieh, D. A parity-breaking electronic nematic phase transition in the spin-orbit coupled metal $Cd_2Re_2O_7$. *Science* **356**, 295-299 (2017).
13. Seyler, K. L. *et al.* Electrical control of second-harmonic generation in a WSe_2 monolayer transistor. *Nat. Nanotech.* **10**, 407-411 (2015).
14. Hong, H. *et al.* Giant enhancement of optical nonlinearity in two-dimensional materials by multiphoton-excitation resonance energy transfer from quantum dots. *Nat. Photon.* **15**, 510-515 (2021).
15. Fu, D. *et al.* Chirality-dependent second-order nonlinear optical effect in 1D organic-inorganic hybrid perovskite bulk single crystal. *Angew. Chem. Int. Ed.* **60**, 20021-20026 (2021).
16. Yuan, C. *et al.* Chiral lead halide perovskite nanowires for second-order nonlinear optics. *Nano Lett.* **18**, 5411-5417 (2018).
17. Burke, B. J., Moad, A. J., Polizzi, M. A. & Simpson, G. J. Experimental confirmation of the importance of orientation in the anomalous chiral sensitivity of second harmonic generation. *J. Am. Chem. Soc.* **125**, 9111-9115 (2003).
18. Petralli-Mallow, T., Wong, T. M., Byers, J. D., Yee, H. I. & Hicks, J. M. Circular dichroism spectroscopy at interfaces: a surface second harmonic generation study. *J. Phys. Chem.* **97**, 1383-1388 (1993).

19. Fichera, B. T. *et al.* Second harmonic generation as a probe of broken mirror symmetry. *Phys. Rev. B* **101**, 241106 (2020).

20. Luo, X. *et al.* Ultrafast modulations and detection of a ferro-rotational charge density wave using time-resolved electric quadrupole second harmonic generation. *Phys. Rev. Lett.* **127**, 126401 (2021).

21. Guo, Z. *et al.* Regulating optical activity and anisotropic second-harmonic generation in zero-dimensional hybrid copper halides. *Nano Lett.* **22**, 846-852 (2022).

22. Zheng, Y. D., Li, J. Q. & Li, C. F. Second harmonic theory of two coupled oscillators in chiral molecular media. *Acta Phys. Sin.* **52**, 372-376 (2003).

23. Elshocht, S. V. *et al.* Direct evidence of the failure of electric-dipole approximation in second-harmonic generation from a chiral polymer film. *J. Chem. Phys.* **107**, 8201-8203 (1997).

24. Maki, J. J., Kauranen, M. & Persoons, A. Surface second-harmonic generation from chiral materials. *Phys. Rev. B* **51**, 1425-1434 (1995).

25. Lam, Y. T. & Thirunamachandran, T. Direct current-induced second harmonic generation by chiral molecules. *J. Chem. Phys.* **77**, 3810-3814 (1982).

26. Lee, S.-H., Goh, J. S. & Cho, D. Origin of the insulating phase and first-order metal-insulator transition in 1T-TaS_2. *Phys. Rev. Lett.* **122**, 106404 (2019).

27. Ritschel, T., Berger, H. & Geck, J. Stacking-driven gap formation in layered 1T-TaS_2. *Phys. Rev. B* **98**, 195134 (2018).

28. Ritschel, T. *et al.* Orbital textures and charge density waves in transition metal dichalcogenides. *Nat. Phys.* **11**, 328-331 (2015).

29. Ma, L. *et al.* A metallic mosaic phase and the origin of Mott-insulating state in 1T-TaS_2. *Nat. Commun.* **7**, 10956 (2016).

30. Chen, X. B., Tian, F. Y., Persson, C., Duan, W. H. & Chen, N.-X. Interlayer interactions in graphites. *Sci. Rep.* **3**, 3046 (2013).
